# Preclinical evaluation of the antineoplastic action of 5-aza-2'-deoxycytidine and different histone deacetylase inhibitors on human Ewing's sarcoma cells

**DOI:** 10.1186/1475-2867-8-16

**Published:** 2008-11-17

**Authors:** Annie Hurtubise, Mark L Bernstein, Richard L Momparler

**Affiliations:** 1 Département de pharmacologie, Université de Montréal, Centre de recherche pédiatrique, and Service d'hématologie-oncologie, Hôpital Sainte-Justine, 3175 Côte Sainte-Catherine, Montréal, Québec, H3T 1C5, Canada; 2 Hematology-Oncology, IWK Health Center and Dalhousie University, Halifax, Nova Scotia, Canada

## Abstract

**Background:**

Most patients with advanced Ewing's sarcoma (EWS) respond poorly to conventional chemotherapy, indicating the need for new treatment approaches. Epigenetic events, such as promoter hypermethylation and chromatin histone deacetylation, silence the expression of tumor suppressor genes (TSGs) and play an important role in tumorigenesis. These epigenetic changes can be reversed by using 5-aza-2'-deoxycytidine (5AZA-CdR), a potent inhibitor of DNA methylation, in combination with an inhibitor of histone deacetylase (HDAC).

**Results:**

Here, we used a clonogenic assay to evaluate the *in vitro *antineoplastic activity of 5AZA-CdR in combination with different HDAC inhibitors on EWS cells. We observed that the HDAC inhibitors, MS-275, trichostatin-A, phenylbutyrate, LAQ824 and depsipeptide, enhanced the antineoplastic action of 5AZA-CdR on EWS cells. The combination of 5AZA-CdR and MS-275 showed marked synergy, and was correlated with significant reactivation of the expression of two TSGs, E-cadherin and tumor suppressor lung cancer-1 (TSLC1), in a EWS cell line.

**Conclusion:**

These results suggest the value of future clinical studies investigating the combination of 5AZA-CdR and MS-275 in patients with advanced EWS.

## Background

Metastatic or recurrent Ewing's sarcoma (EWS) does not respond well to standard chemotherapy [1], suggesting the need for new therapeutic approaches for the treatment of this malignancy. The silencing of tumor suppressor genes (TSGs) by aberrant DNA methylation plays an important role in tumorigenesis [2]. Since this epigenetic change is reversible, it is a potential target for chemotherapeutic intervention. 5-Aza-2'-deoxycytidine (Decitabine, Dacogen, 5AZA-CdR), a potent inhibitor of DNA methylation, has been shown to reactivate the expression of silenced TSGs [3]. 5AZA-CdR has been approved for the treatment of hematological malignancies [4,5]. However, its antitumor activity is still under investigation. Certain genes that inhibit cellular growth can also be silenced by the deacetylation of chromatin-bound histones, which yields a compact chromatin configuration that is unfavorable for transcription [6]. Histone deacetylase (HDAC) inhibitors can reverse this process to produce an antitumor effect [7]. In addition to reactivation of genes that inhibit tumor growth, HDAC inhibitors can produce cell cycle arrest and induce apoptosis [8]. These inhibitors are currently under clinical investigation in patients with different types of malignancies [9].

Research has shown that the "cross-talk" between DNA methylation and histone modifications in chromatin can synergistically re-activate TSGs [10], suggesting that it might be useful to investigate the use of 5AZA-CdR in combination with HDAC inhibitors for tumor therapy. We previously reported that 5AZA-CdR plus depsipeptide (depsi) or phenylbutyrate (PB) showed synergistic antineoplastic action against breast carcinoma cells [11] and lung carcinoma cells [12], respectively. We also investigated the ability of 5AZA-CdR and MS-275 to reactivate two TSGs, E-cadherin (ECAD) [13] and tumor suppressor lung cancer-1 (TSCL1)[14], in EWS cells. Here, we evaluated the *in vitro *antineoplastic activity of 5AZA-CdR in combination with different HDAC inhibitors: depsi, PB, trichostatin-A (TSA), LAQ824 (LAQ) and MS-275 in EWS cells. Our results revealed that all of the tested HDAC inhibitors enhanced the antineoplastic action of 5AZA-CdR on EWS cells.

## Methods

### Material

5AZA-CdR was obtained from Pharmachemie (Haarlem, Netherlands). Depsi (FR901228) was obtained from Fujisawa Pharmaceutical (Osaka, Japan). LAQ was kindly provided by Novartis Pharmaceuticals Inc. (East Hanover, NJ). TSA was obtained from Wako BioProducts (Richmond, VA). MS-275 was kindly provided by Schering AG (Berlin, Germany). PB was procured from Triple Crown America Inc. (Perkasie, PA). The human TC71 and TC32 EWS cell lines were kindly provided by Dr Jeffrey A. Toretsky (Lombardi Comprehensive Cancer Center, Georgetown University, Washington, DC). The cells were cultivated as monolayer in RPMI 1640 medium (Life Technologies, Burlington, Ontario) with 10% heat-inactivated fetal calf serum (Wisent, St-Bruno, Quebec) at 37°C with 5% CO_2 _atmosphere.

### Clonogenic assay

The loss of clonogenicity of TC71 and TC32 EWS cell lines was assessed after drug exposure by placing 100–250 cells in each well of a six-well 35 mm dish. The next day different concentrations of 5AZA-CdR and/or HDAC inhibitors: depsi, TSA, PB, MS-275 or LAQ were added at indicated concentrations for 48 h. The cells were washed with drug-free medium and were incubated for an additional 7–11 days and then stained with 0.5% methylene blue in 50% methanol. The colonies (> 500 cells) were counted.

### Inhibition of DNA synthesis assay

The inhibition of DNA synthesis by 5AZA-CdR and/or HDAC inhibitors was measured by the incorporation of radioactive thymidine into DNA. Aliquots of ~10^4^cells in 2 ml of medium were placed in each well of a six-well 35 mm dish. The next day, the cells were exposed to the different concentrations of 5AZA-CdR and/or HDAC inhibitors as indicated above. Then, at 48 h, 0.5 μCi of radioactive tritium-labeled thymidine (6.7 Ci/mmol, ICN Biomedicals, Irvine, CA) was added to the medium for an additional 24 h. The cells were then trypsinised, suspended in 0.9% NaCl, placed on a GF/C 25 mm glass fiber filter disc, washed with cold 0.9% NaCl, 5% cold trichloroacetic acid and ethanol. The filters were dried, placed in EcoLite scintillation fluid (ICN Biomedicals) and the radioactivity was measured with a scintillation counter.

### Isolation of RNA and RT-PCR analysis

In order to study the reactivation of gene in EWS cell lines, we treated cells with 5AZA-CdR (100 ng/ml) and/or MS-275 (250 ng/ml) for 72 h. Cells were harvested 24 h after the removal of the drugs and total RNA was isolated using RNeasy Mini Kit (Qiagen, Mississauga, Ontario). For cDNA synthesis, total RNA was reverse-transcribed using OmniScript RT kit (Qiagen). The reaction was performed at 37°C for 1 h followed by 5 min at 93°C to inactivate the enzyme. PCR amplifications were performed using HotStar Taq Polymerase (Qiagen) and specific primers spanning different exons for ECAD, TSLC1 and 18S ribosomal RNA. For ECAD (GenBank NM_004360), the primers were sense 5'-CAATCCCACC ACGTACAAG-3' and antisense 5'-CTGGGCAGTGTAGGATGTGA-3'. The length of the PCR product of ECAD was 410 bp. For TSLC1 (GenBankNM_014333), the primers were sense 5'-GGGCAGAATCTGTTTA CGAAAGA-3' and antisense 5'-TCGGTATAGAGCTGGCAAAAGTA-3'. The length of the PCR product of TSLC1 was 257 bp. The human 18S ribosomal RNA gene (GenBank X03205) was amplified as an internal control using as sense primer 5'-TCGATGGTAG TCGCCGTGCCTA-3' and antisense 5'-CTGCTGCCTTCCTTGGATGTGGTA-3'. The length of the PCR product of 18S ribosomal RNA was 110 bp. Samples were amplified in a thermocycler under the following conditions. For ECAD, the PCR conditions were 5 min at 95°C, 15 s at 94°C, 15 s at 58°C and 15 s at 72°C, for 5 cycles. Then, the annealing temperature was lowered at 56°C for 35 more cycles. For TSLC1, the PCR conditions were 5 min at 95°C, 30 s at 94°C, 15 s at 56°C and 15 s at 72°C, for 5 cycles. Then, the annealing temperature was lowered at 54°C for 34 more cycles. For 18S ribosomal RNA, the PCR conditions were 5 min at 95°C, 45 s at 94°C, 30 s at 60°C and 30 s at 72°C, for 5 cycles. Then, the annealing temperature was lowered at 58°C for 11 more cycles. For each gene, the number of cycle chosen as to be in the exponential phase of DNA amplification. The PCR products were electrophoresed on 2% agarose gel and detected by ethidium bromide staining. The measurement of the absolute concentration of amplified DNA was obtained with the Agilent 2100 Bioanalyzer (Agilent Technologies, Palo Alto, CA) as described previously.[11] This latter method, which is very sensitive, uses capillary electrophoresis and fluorescent detection to measure both the size and quantity of DNA.

### Data analysis

The data are the mean values ± SD for n ≥ 3. Differences between groups were analyzed using one-way ANOVA test coupled with a Tukey-Kramer test, by comparing the result of each drug alone with the results of the combination of both agents. The critical level of significance was set at p ≤ 0.05.

## Results

**Figure 1:**
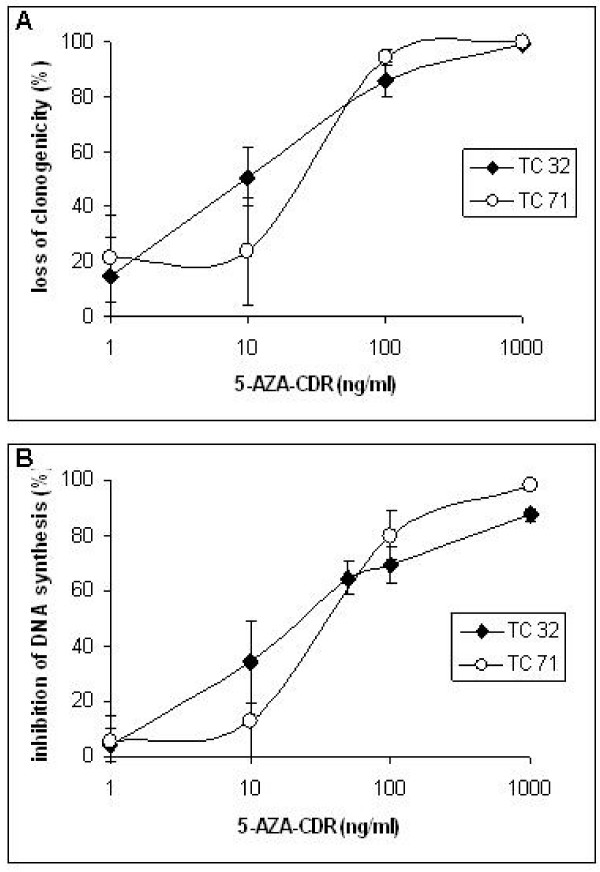
**Effect of different concentrations of 5AZA-CdR for 48 h exposure on loss of clonogenicity (A) and for 72 h exposure on inhibition of DNA synthesis (B) for TC32 and TC71 EWS cells.** Data shown are mean values ± S.D., n ≥ 3.

The effects of different concentrations of 5AZA-CdR on loss of clonogenicity and on the inhibition of DNA synthesis in TC71 and TC32 EWS cell lines are shown in Figure 1A and 1B, respectively. The concentration of 5AZA-CdR that produced about 50% loss of clonogenicity (IC_50_) following a 48 h exposure was in the range of 10 ng/ml for the TC32 cell line and 30 ng/ml for the TC71 cell line. The IC_50 _values for inhibition of DNA synthesis following a 72 h exposure of 5AZA-CdR were in the same range as the IC_50 _values for loss of clonogenicity for both ES cell lines.

**Figure 2:**
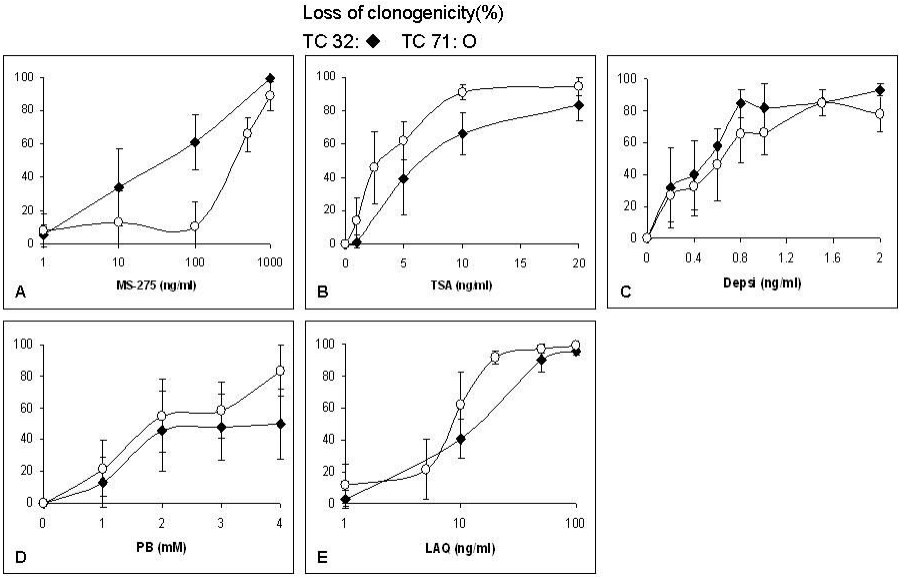
**Effect of different concentrations of HDAC inhibitors for 48 h exposures on loss of clonogenicity for TC32 and TC71 EWS cells.** Data shown are mean values ± S.D., n ≥ 3.

**Figure 3:**
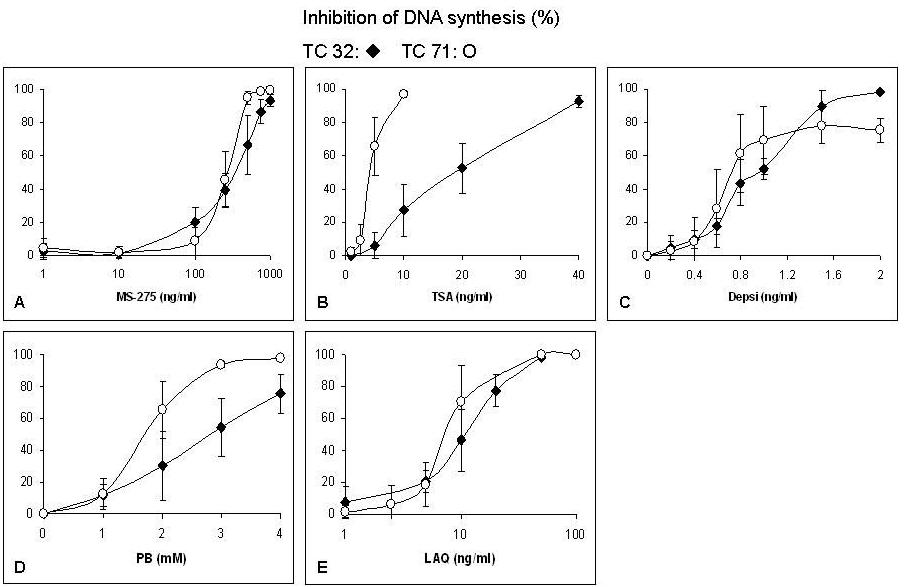
**Effect of different concentrations of HDAC inhibitors for 72 exposures on inhibition of DNA synthesis for TC32 and TC71 EWS cells.** Data shown are mean values ± S.D., n ≥ 3.

For the HDAC inhibitors, the IC_50 _values for loss of clonogenicity for the TC32 and TC71 cell lines, respectively, were: ~30 and 400 ng/ml for MS-275 (Figure 2A), ~7 and 2 ng/ml for TSA (Figure 2B), ~0.5 and 0.6 ng/ml for depsi (Figure 2C), ~2 mM for PB (Figure 2D) and ~12 and 8 ng/ml, for LAQ (Figure 2E). For the inhibition of DNA synthesis the IC_50 _values for the HDAC inhibitors for the TC32 and TC71 cell lines, respectively, were: ~400 ng/ml for MS-275 (Figure 3A), ~20 and 2 ng/ml for TSA (Figure 3B), ~1 and 0.8 ng/ml for depsi (Figure 3C), ~3 and 2 mM for PB (Figure 3D) and ~10 and 8 ng/ml for LAQ (Figure 3E).

**Figure 4:**
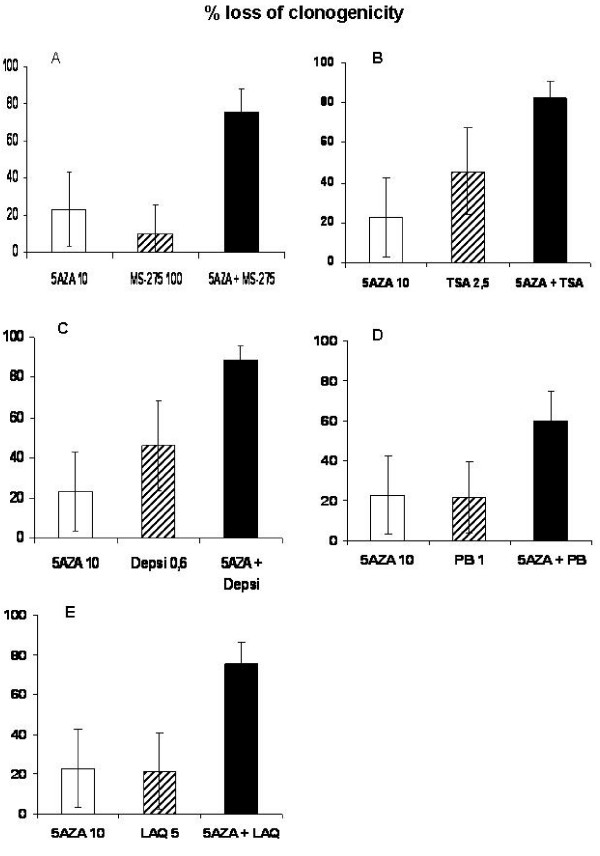
**Effect of 48 h exposure of 5AZA-CdR (5AZA) (10 ng/ml) and/or HDAC inhibitors on loss of clonogenicity of TC71 EWS cells.** A) MS-275 (100 ng/ml), B) TSA (2.5 ng/ml), C) Depsi (0.6 ng/ml), D) PB (1 mM), E) LAQ (5 ng/ml). Data shown are mean values ± S.D., n ≥ 3. Statistical analysis: 5AZA + HDAC inhibitor > 5AZA or HDAC inhibitor p < 0.01.

The objective of this study was to determine if 5AZA-CdR in combination with HDAC inhibitors would show enhanced antineoplastic activity against the EWS cells. In our clonogenic assay 5AZA-CdR plus the HDAC inhibitors TSA, PB, LAQ, depsi and MS-275 showed a significant enhancement of their antineoplastic effect as compared to each agent alone (p < 0.01) for the TC71 EWS cells (Figure 4). A clear synergistic interaction was observed between 5AZA-CdR and MS-275 or LAQ as defined by Valeriote and Lin [15].

**Figure 5:**
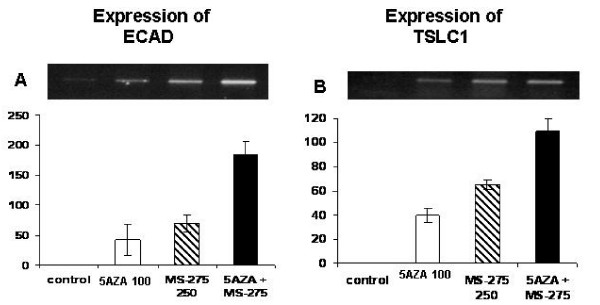
**Effect of 5AZA-CdR (5AZA) (100 ng/ml) and/or MS-275 (100 ng/ml) after 72 h exposure on the induction of expression of ECAD (A) and TSLC1 (B) in TC71 EWS cells as determined by RT-PCR.** The amplified cDNA was analyzed by electrophoresis on agarose (top) and quantitated by Agilent Bioanalyzer 2100 (bottom). The cDNA of 18S ribosomal RNA was used as an internal standard. Vertical axis values are for relative expression. Data shown are mean values ± S.D, n ≥ 3. Statistical analysis for (A) and (B): 5AZA or MS-275 versus 5AZA + MS-275: p < 0.01.

The antineoplastic activity of 5AZA-CdR is related to its reactivation of TSGs silenced by aberrant methylation. Since the combination of 5AZA-CdR and MS-275 produced the most potent synergistic antineoplastic interaction on the TC71 EWS cells, we investigated their reactivation of the TSGs, ECAD and TSLC1 in this cell line (Figure 5). We observed that the combination of 5AZA-CdR and MS-275 produced a synergistic reactivation of the expression of these genes as compared to either agent alone.

## Discussion

There is an urgent need to develop new approaches for the chemotherapy of advanced EWS. The inhibitor of DNA methylation, 5AZA-CdR, is an interesting agent to investigate for tumor therapy, since it can reactivate silenced TSGs [3]. This epigenetic agent has proven effective in patients with hematological malignancies [4,5], and has shown some promising activity in patients with advanced lung cancer [16]. The HDAC inhibitors are another class of epigenetic agents that are of interest in the context of cancer therapy. These inhibitors convert chromatin to an open, transcription-facilitating conformation [6], and have been shown to have diverse actions on tumor cells, including activation of growth-inhibiting genes, induction of apoptosis, inhibition of cell cycle progression, and inhibition of human tumor xenografts in nude mice [8,17,18]. Several HDAC inhibitors are currently under clinical investigation in patients with solid tumors [9].

In this study, we first evaluated the single-agent antineoplastic action of 5AZA-CdR and five different HDAC inhibitors on human TC71 and TC32 EWS cell lines. In assays on loss of clonogenicity and inhibition of DNA synthesis, we observed good dose-response curves with 5AZA-CdR and all of the HDAC inhibitors. Both classes of epigenetic agents showed potential as single agents for the treatment of EWS.

Since a landmark study showed that an inhibitor of DNA methylation in combination with an HDAC inhibitor produced a synergistic reactivation of TSGs in neoplastic cells [10], we next investigated whether 5AZA-CdR in combination with HDAC inhibitors produces an additive or synergistic antineoplastic effect on EWS cells. In the clonogenic assay, we observed that 5AZA-CdR in combination with different HDAC inhibitors (TSA, LAQ, PB, Depsi and MS-275) produced an additive or synergistic antineoplastic interaction against TC71 EWS cells. We previously reported a synergistic antineoplastic interaction between 5AZA-CdR and some of these HDAC inhibitors for breast and lung tumor cell lines [11,12].

## Conclusion

In particular, the combination of 5AZA-CdR and MS-275 showed a marked synergistic interaction with respect to antineoplastic activity against EWS cells. This combination also produced a synergistic reactivation of two TSGs: ECAD and TSLC1. A previous report showed that MS-275 has significant *in vitro *and *in vivo *antitumor activity against EWS [17,18], and MS-275 showed promising results in a phase I study on patients with EWS [19]. These previous findings and our present results combine to suggest that 5AZA-CdR and MS-275 may be a good combination of epigenetic agents to investigate in patients with advanced EWS.

## Competing interests

The authors declare that they have no competing interests.

## Authors' contributions

AH planned, performed the experimental work and wrote the manuscript. RLM planned the experimental work, evaluated the data and revised the manuscript. MLB evaluated the data and revised the manuscript.
